# Intelligence

**Published:** 1809-10

**Authors:** 


### INTELLIGENCE.

A Correspondent of Nicholson's Journal, has discovered a process for obtaining from ginger, an acid, which he proposes to call, zingiberic. One ounce of the best white ginger was infused two or three days in six ounces of nitrous acid; after which, rather more than an equal quantity of water was added, and the whole was kept at the heat of 212°, adding water to supply the loss loss by evaporation, till the nitrous smell had disappeared. Carbonate of lead was then added to saturation, and the solution filtered, after which the lead was precipitated by sulphuric acid, and a second filtration was made. By evaporating the filtered liquor, an acid similar in appearance to short white pieces of raw silk was obtained, which oxidates zinc and iron, and dissolves potash, soda, and ammonia, barytes, strontian, lime, magnesia, and the oxides of zinc, iron, lead, and copper. The zingiberic acid differs from the sulphuric, sulphurous, carbonic, oxalic, tartarous, citric, mucous, succinic, and camphoric acids, in forming a soluble salt with barytes and lime; from the nitric, nitrous, muriatic, acetic, acetous, sebacic, malic, and prussic, by remaining in the solid form at 212°; from the benzoic and suberic, by its greater solubility; and it does not, like gallic acid, precipitate copper of a brown colour.

The following very curious case we should have recorded earlier, but that we expected it to appear *first* from a gentleman of the Hospital in the transactions of some Society.—A sailor, at Guy's Hospital, who had frequently been in the practice of swallowing pocket knives, shut, either out of bravado, or for trifling wagers. It appears that he was first induced to make so hazardous an experiment by having believed that he had seen another do it in America; and when laughed at for his credulity, he thought there was no argument so convincing as performing the exploit before his incredulous audience. The success of his first experiment emboldened him to repeat his story, and confirm it by proof. He observed, however, that the three first which he swallowed, passed through him not in the order in which they were swallowed, but reversely, and this led him to believe that there was plenty of room for them to pass each other, and that there was no danger of any stoppage or interuption in their progress through the intestinal canal. The reader will be anxious to know of what size or kind the knives were, he will most probably conclude they were small penknives; they were, on the contrary, such knives as are usually carried by sailors and labourers to cut their food with. It appears that he had continued in this practice for several years without experiencing any material inconvenience; no long time, however, before his admission into the Hospital, he had swallowed one which took a position directly across the stomach, and in that position it became confined, and produced the disease which ultimately proved fatal. On examination after death, not only the fatal instrument, but thirteen others were found within the stomach and intestines, in all of which the horn of the handles was nearly digested, and the iron and steel of some considerably corroded, probably by the acid which he had taken with a view to dissolve the one which caused his death.

Dr. Ewell, of Washington, in America, has given an account of the successful internal exhibition of the acetate, or sugar of lead, in several diseases, particularly in profuse hæmorrhage, and in cases of salivation. He is also of opinion, that it is worthy of a trial in dysentery, at least after evacuants have been used.

M. Par-

M. PARMENTIER, whose labours are always directed to some useful end, has made public a new method of preparing the extract of opium, which appears far superior to all those hitherto known. It takes from that substance the smell by which it is distinguished, and which is always in proportion to its malignant qualities. The manner of preparing 24 ounces of opium is as follows: Macerate it in rain-water for five days: then boil for a quarter of an hour with two pounds of pulverized charcoal: strain and clarify with white of egg, and, by evaporation, you will obtain twelve ounces of extract.

M. LENORMAND has succeeded in producing a fine colourless varnish with copal. All copal is not fit for this purpose; and to ascertain such pieces as are good, each must be taken separately, and a single drop of pure essential oil of rosemary, not altered by keeping, must be let fall on it. Those pieces which soften at the part that imbibes the oil, are good; reduce them to powder, which sift through a very fine hair sieve, and put it into a glass, on the bottom of which it must not lie more than a finger's breadth thick. Pour upon it essence of rosemary to a similar height; stir the whole for a few minutes, when the copal will dissolve into a viscous fluid. Let it stand for two hours, and then pour gently on it two or three drops of very pure alcohol, which distribute over the oily mass, by inclining the bottle in different directions with a very gentle motion. Repeat this operation by little and little, till the incorporation is effected, and the varnish reduced to a proper degree of fluidity. It must then be left to stand a few days, and when very clear be decanted off. This varnish, thus made without heat, may be applied with equal success to pasteboard, wood, and metals, and takes a better polish than any other. It may be used on paintings, the beauty of which it greatly heightens.

M. FOURNIER has invented an apparatus for determining, with precision, the quantity of spirit contained in any liquid, to which he gave the name of alcohometer. This instrument is composed of a glass tube, six or seven inches long, and placed vertically on a cap of copper, and having a graduated bar of the same metal attached to its centre. At the place where the bar enters the tube adjusted to its base, there is a screw, by which it is hermetically closed, and which prevents the liquid to be analysed from spilling. This little apparatus stands upon three legs: at the foot is a lamp with spirit of wine, placed under the copper cap, and directly beneath the bar, to heat it quickly. On one of the legs is a moveable ferrule with a damper, for the purpose of moderating at pleasure the action of the flame, and thus preventing the liquid in the tube from running over.

---

MEDICAL THEATRE, ST. BARTHOLOMEW'S HOSPITAL.

The following Courses of Lectures will be delivered in this Theatre during the ensuing winter:—On the Theory and Practice of Medicine, by Dr. Powell; on Anatomy and Physiology, by Mr. Abernethy;

Abernethy; on the Theory and Practice of Surgery, by Mr. Abernethy; on Midwifery, by Dr. Thynne; on Chemistry, by Dr. Hue; on Comparative Anatomy and Physiology, by Mr. Macartney; Anatomical Demonstrations, by Mr. Lawrence. The Lectures will begin on Monday, October 2, at Two o'clock. Further particulars may be known by applying to Mr. Wheeler, Apothecary to the Hospital.

Mr. CARLISLE will commence his usual Course of Lectures on the Art and Practice of Surgery, on Tuesday, October 11, at Eight o'clock, P. M. at his House in Soho Square. These Lectures are intended to comprise such Pathological Remarks as are applicable to the Theory and Management of Surgical Practice. Other particulars may be known by inquiry.

Dr. SATTERLEY's Course of Clinical Instruction will begin the first week in November, at the Middlesex Hospital; the attendance on the patients will be continued daily, and Lectures will be given once a week, or oftener, when it may be necessary, at eleven o'clock. Mr. CARTWRIGHT, Assistant Surgeon to the Hospital, will undertake such occasional demonstrations of Morbid Anatomy, as may be required for the illustration of the respective cases. The objects of the Course will also be extended to such remarkable peculiarities in the diseases of Children, as may occur in the Foundling Hospital. Dr. YOUNG will begin his Elementary Lectures on Chemistry, Physiology, the Practice of Physic, and the Materia Medica, about the middle of December; he will deliver them on Mondays, Wednesdays, and Fridays, at Seven o'clock in the evening, throughout the season.

Mr. MOOR, Surgeon Dentist to her Royal Highness the Duchess of York, will commence a Course of Lectures on the Structure and Diseases of the Teeth, on the first Thursday in November, in which will be explained the Complete Practice of the Dentist. Further Particulars may be known at his house, No. 6, Palsgrave Place, Temple.

